# Development of Anti-inflammatory Probiotic *Limosilactobacillus reuteri* EFEL6901 as Kimchi Starter: *in vitro* and *In vivo* Evidence

**DOI:** 10.3389/fmicb.2021.760476

**Published:** 2021-11-25

**Authors:** Hee Seo, Hyunbin Seong, Ga Yun Kim, Yu Mi Jo, Seong Won Cheon, Youngju Song, Byung Hee Ryu, Hee Kang, Nam Soo Han

**Affiliations:** ^1^Brain Korea 21 Center for Bio-Resource Development, Division of Animal, Horticultural, and Food Sciences, Chungbuk National University, Cheongju, South Korea; ^2^Department of Biomedical Science and Technology, Graduate School, Kyung Hee University, Seoul, South Korea; ^3^Fresh Food Research Division, Food BU, Daesang Corporation Research Institute, Icheon, South Korea; ^4^Humanitas College, Kyung Hee University, Yongin, South Korea

**Keywords:** *Limosilactobacillus reuteri*, probiotic, kimchi starter, inflammatory bowel disease, DSS-induced colitis, anti-inflammation

## Abstract

The use of probiotic starters can improve the sensory and health-promoting properties of fermented foods. In this study, we developed an anti-inflammatory probiotic starter, *Limosilactobacillus reuteri* EFEL6901, for use in kimchi fermentation. The EFEL6901 strain was safe for use in foods and was stable under human gastrointestinal conditions. In *in vitro* experiments, EFEL6901 cells adhered well to colonic epithelial cells and decreased nitric oxide production in lipopolysaccharide-induced macrophages. In *in vivo* experiments, oral administration of EFEL6901 to DSS-induced colitis mice models significantly alleviated the observed colitis symptoms, prevented body weight loss, lowered the disease activity index score, and prevented colon length shortening. Analysis of these results indicated that EFEL6901 played a probiotic role by preventing the overproduction of pro-inflammatory cytokines, improving gut barrier function, and up-regulating the concentrations of short-chain fatty acids. In addition, EFEL6901 made a fast growth in a simulated kimchi juice and it synthesized similar amounts of metabolites in nabak-kimchi comparable to a commercial kimchi. This study demonstrates that EFEL6901 can be used as a suitable kimchi starter to promote gut health and product quality.

## Introduction

Inflammatory bowel disease (IBD) is a chronic disease characterized by relapsing inflammation of the gastrointestinal (GI) tract, and includes Crohn’s disease and ulcerative colitis under this definition (Witkowski et al., 2018). The pathogenesis of IBD is unknown and it has been suggested that the environmental exposure of a subject causes changes in the intestinal microbiota and subsequently changes the epigenetic imprinting of the mucosa and the associated immune system (Rogler and Vavricka, 2015). In particular, altered gut bacteria and modified bacterial metabolic pathways are two important factors in the initiation and progression of IBD, and many investigations favor the altered microbiome as the primary cause of inflammatory reactions (Ganji-Arjenaki et al., 2017). Studies have shown that the therapeutic use of antibiotics for IBD causes dysbiosis and changes in the microbial population, as well as dysregulation of the immune response, resulting in intolerance to treatment and secondary infection with *Clostridium difficile* (Sheehan et al., 2015; Nitzan, 2016). Meanwhile, microbial-based therapeutics, such as probiotics, have received considerable attention for the treatment and prevention of IBD due to their perceived natural and safe aspects (Jakubczyk et al., 2020). Probiotics can help alleviate IBD by enhancing mucosal barrier function, inhibiting pathogenic bacteria, modulating immune responses, and altering gut microbiota composition (Witkowski et al., 2018).

Fermented foods can be effective delivery vehicles of probiotic bacteria, and the fermented matrices and metabolites can also increase the probiotic potential of those strains (Tamang et al., 2020; Sauer and Han, 2021). Kimchi is a traditional Korean fermented vegetable dish made with salted cabbage, radish, and cucumber, and various spices (Jang et al., 2015). The use of starter cultures in kimchi manufacture has several advantages, such as consistent quality, improved sensory characteristics, extended shelf-life, and functional properties in the kimchi product (Chang and Chang, 2010; Jo et al., 2015). Since kimchi is fermented via lactic acid bacteria and often consumed with daily meals, it is regarded as one of the best food matrices to deliver probiotics (Seo et al., 2021). Therefore, it is necessary to develop a kimchi probiotic that can be used not only as a starter in kimchi, but also as a microorganism to protect humans from diseases such as IBD. For this purpose, various lactic acid bacteria have been developed as probiotic starters, including *Bifidobacterium* spp. producing conjugated linoleic acid (Raimondi et al., 2016) and *Lactobacillus* spp. producing γ-aminobutyric acid (GABA) (Das and Goyal, 2015). However, these probiotic candidates exhibit little or no growth in kimchi; thus, there are few commercialized examples of probiotic starters to date.

In our previous study, we discovered that *Limosilactobacillus reuteri* species could be considered as potential candidates for kimchi starters, among 17 other species of probiotic type strains tested (Seo et al., 2021). Thereby, the goal of this study was to develop an anti-inflammatory probiotic starter, *Limosilactobacillus reuteri*, for kimchi fermentation. For this purpose, we isolated various strains of *L. reuteri* from different fermented foods and microbial sources, and compared their probiotic and starter properties using serial *in vitro* tests. Among the strains tested, we obtained a suitable probiotic candidate, *L. reuteri* EFEL6901, which showed not only anti-inflammatory and probiotic activities, but also fast growth rate in simulated kimchi juice (SKJ) with comparable levels to the available commercial strains. We tested its safety, stability in the GI tract, antioxidant activity, and anti-inflammatory activity *in vitro* and *in vivo.* In addition, we tested the fermentation profiles of EFEL6901 in SKJ and nabak-kimchi to evaluate its suitability as kimchi starter.

## Materials and Methods

### Bacterial Strains and Growth Conditions

The bacterial strains used in this study are listed in Supplementary Table 1. *L. reuteri* EFEL6901 was isolated from human feces and deposited in the Korean Agricultural Culture Collection (KACC, Korea, accession number KACC81105BP, Wanju, Korea). For probiotic activity tests, *Lactiplantibacillus plantarum* WCFS1 and *Lacticaseibacillus rhamnosus* GG were used as positive controls. To analyze the characteristics of kimchi starter, a commercial kimchi starter, *Leuconostoc mesenteroides* DRC1506, which was kindly provided by Daesang FNF (Daesang, Icheon, Korea), was used as a positive control. All strains were stored in 15% glycerol solution at −80°C. Prior to the experiment, the strains were cultured in MRS broth (BD Difco, Franklin Lakes, NJ, United States) for 24 h under optimal culture conditions. When the final concentration reached 10^9^ CFU/mL, bacterial cells were washed twice with sterile phosphate-buffered saline (PBS; pH 7.2) and used in the experiment.

### Evaluation of Safety and Stability

#### Safety Test

The presence of genes that produce biogenic amines in the bacterial strains was analyzed by multiplex PCR, as described by O’Sullivan et al. (2015). The *hdc* (histidine decarboxylase) and *tyrdc* (tyrosine decarboxylase) genes were amplified using the following primer pairs: *hdc*: HDC3 (5′- GATGGTATTGTTTCKTATGA- 3′) and HDC4 (5′- CAAACACCAGCATCTTC- 3′); *tyrdc*: TD2 (5′- ACATAGTCAACCATRTTGAA- 3′) and TD5 (5′-CAAATGGAAGAAGAAGTAGG- 3′); 16S rRNA gene: 27F and 1492R. In the multiplex PCR, each biogenic amine gene was amplified simultaneously with 16S rRNA gene in a PCR tube by adding each corresponding primer set. Genomic DNA from bacterial cells was used as the template in the PCR reaction. The amplification program was as follows: 95°C for 5 min, followed by 32 cycles of 95°C for 45 s, 58°C for 45 s, and 72°C for 75 s, with a final extension at 72°C for 5 min. *L. reuteri* ATCC 23272 and *Enterococcus faecalis* KCCM 11729 were used as positive controls for the detection of *hdc* and *tyrdc* genes, respectively.

The hemolytic activity of the bacterial strains was determined according to the protocol described by Ryu and Chang (2013). Bacterial cells were inoculated in BHI agar plates supplemented with 7% horse blood (MB CELL, Seoul, Korea) and incubated for 24 h at 37°C under anaerobic conditions. After incubation, hemolytic activity was determined by observing a blood lysis halo around the colonies (β-hemolysis) on the medium. *Listeria monocytogenes* was used as a positive control for β-hemolysis.

#### Acid and Bile Salt Tolerance Assay

Resistance to acidic conditions was tested using the method proposed by Conway et al. (1987). Lactic acid bacteria were cultured in MRS medium overnight and when the final concentration reached 10^8^ CFU/mL harvested by centrifugation at 6,000 × *g* for 10 min. Cells were washed twice with PBS (pH 7.2) and resuspended in equal volumes of PBS adjusted to pH 3.0 and 2.5 with HCl. Acid tolerance was evaluated by spreading cells on MRS agar medium after 0, 90, and 180 min at 37°C, followed by counting viable cells after incubation at 37°C for 48 h (Maragkoudakis et al., 2006). Bile acid tolerance was evaluated by suspending the cells in PBS solution containing 0.3% (w/v) bile salt (Sigma, St Louis, MO, United States) and incubating at 37°C for 90 and 180 min (Gilliland et al., 1984). After incubation at 37°C for 48 h, the colonies were counted on MRS agar medium, and the survival rate was calculated.

### *In vitro* and *ex vivo* Cell Test

#### Adhesion to HT-29 Cells

The adhesion assay was performed as described by Messaoudi et al. (2012). HT-29 cells, a human colonic epithelial cell line, were obtained from the Korean Cell Line Bank (KCLB; Seoul, Korea) and grown in Dulbecco’s Modified Eagle’s Medium (DMEM; Hyclone, Logan, UT, United States) supplemented with 10% fetal bovine serum (FBS; Hyclone), and 1% each of 10,000 U/mL penicillin and 10 mg/mL streptomycin (Hyclone) in 0.85% NaCl. HT-29 cells were seeded in 24-well tissue culture plates (2 cm^2^ per well) at a density of 4.7 × 10^5^ cells per well. Once the culture reached 80% confluency, the medium was changed to one without antibiotics. Live bacteria (10^8^ CFU/mL, washed twice in PBS, numerated by CFU counting, ratio 100 bacteria:1 HT-29 cell, MOI 100) were inoculated to 10^6^ cells in DMEM culture medium without antibiotics and incubated at 37°C in 5% CO_2_ for 2 h. After incubation, non-adherent bacteria were removed by washing twice with PBS. Cells with adhered bacteria were treated with a detachment solution containing 0.1% Triton X-100 and 0.1% trypsin-EDTA (Sigma) for 15 min. To calculate the number of adherent bacteria, the suspensions of the detached cells were plated onto MRS agar and incubated at 37°C for 48 h. The adhesion ability was estimated using the formula (the adhered bacteria/100 cells), where HT-29 cells were counted with hemocytometer (Thoma, Hirschmann, Germany).

#### Antioxidative Activity Assay

Antioxidative activity of the two fractions of bacterial cells was measured by the DPPH inhibition assay as proposed by Das and Goyal (2015). For preparation of intact cells and cell-free extracts, bacteria were pre-cultured for 12 h, grown in the main culture for 12 h, and the optical density at 600 nm (OD_600_) was adjusted to 1.0 by dilution. The bacterial cells were washed twice and resuspended in 0.85% saline solution to make intact cells. For cell-free extract, sonication was performed in a sonicator (VP-050N; Taitec Corp., Saitama, Japan) for 10 min (5 s on/5 s off pulse at 35% amplitude), and the cell debris was removed by centrifugation (10,000 × *g* at 4°C for 5 min). The ethanolic DPPH solution (100 μL, 0.4 mmol/L) was mixed vigorously with 100 μL of bacterial sample or molecular-grade water (control) and incubated at 37°C in the dark for 30 min. The absorbance of the mixture was measured at 517 nm using a microplate reader (BioTek, Winooski, VT, United States).

#### Measurement of Nitric Oxide (NO) Production

The effect of the EFEL6901 strain on the production of NO was determined in LPS-induced RAW 264.7 cells using the Griess reaction (Yu et al., 2019). For the preparation of heat-killed bacterial cells, the absorbance of the strains was adjusted to OD_600_ of 1.0 (5 × 10^8^ cells/mL). After centrifugation at 10,000 × *g* for 5 min, the cell pellets were washed twice with sterile PBS (pH 7.2) and suspended in DMEM. Bacterial cells were heated at 95°C for 30 min. The murine macrophage cell line, RAW 264.7, was obtained from the Korean Cell Line Bank and maintained in DMEM containing 10% FBS and 1% penicillin-streptomycin at 37°C in a humidified atmosphere of 5% CO_2_. Cells were sub-cultured and plated at 80–90% of confluency. Cells (5 × 10^5^ cells/mL) were seeded in 96-well plates and incubated for 24 h. Cells were treated with LPS (1 μg/mL), followed by the addition of heat-killed bacterial cells for 24 h. After incubation, the culture supernatant from each well was mixed with an equal volume of Griess reagent (Sigma) and placed in the dark for 15 min at room temperature. The absorbance of each well was measured at 540 nm wavelength. Nitrite concentration was calculated using dilutions of sodium nitrite as a standard, and fresh culture medium was used as the blank control.

#### Measurement of Nitric Oxide Synthase (iNOS) and Cyclooxygenase-2 (COX-2) mRNA Expression Level

The effect of the EFEL6901 strain on the pro-inflammatory transcriptome was assessed in LPS-induced RAW 264.7 cells using RT-qPCR. RAW264.7 cells (1 × 10^6^ cells/well) were seeded in 6-well plates and incubated for 24 h. Cells were treated with LPS (1 μg/mL) with or without heat-killed bacterial cells. After incubation, cells were washed twice with sterile PBS (pH 7.2), and total RNA was extracted using the TRIzol RNA isolation reagent (Invitrogen, Waltham, MA, United States) according to the manufacturer’s protocol. The total RNA was quantified by absorbance at 260 nm using a nanodrop SpectraMax 190 (Molecular devices, San Jose, CA, United States). An adequate amount of RNA was reverse-transcribed into cDNA using the LeGene Express 1st Strand cDNA Synthesis System Kit (LeGene Biosciences, San Diego, CA, United States). Real-time PCR was conducted using a CFX96 real-time PCR system (Bio-Rad, Hercules, CA, United States). The mixtures containing synthesized cDNA, 10 pmol of specific primers, and PCR Master Mix containing SYBR Green Mix were amplified as follows: 95°C for 5 min, followed by 40 cycles at 95°C for 15 s, 60°C for 30 s, and a final extension at 60°C for 30 s. Single-product amplification was verified by melting curve analysis at the end of the experiment. The results were analyzed after normalization with GAPDH as the reference gene. Relative expression levels of target genes were calculated using the ΔΔCt method (Livak and Schmittgen, 2001). Primer pairs for cytokine mRNA expression levels are GAPDH; (Forward) 5′-TTGTCTCCTGCGACTTCAACA-3′ (Reverse) 5′-GCTGTAGCCGTATTCATTGTCATA-3′, iNOS; (Forward) 5′-ACCATGGAGCATCCCAAGTA-3′ (Reverse) 5′-CCATGTACCAACCATTGAAGG-3′, COX-2; (Forward) 5′-AGCATTCATTCCTCTACATAAGC-3′ (Reverse) 5′-GTAACAACACTCACATATTCATACAT-3′, TNF-α; (Forward) 5′-ATGATCCGCGACGTGGAA-3′ (Reverse) 5′-ACCGCCTGGAGTTCTGGA-3′, IL-1β; (Forward) 5′-GTTGACGGACCCCAAAAGAT-3′ (Reverse) 5′-CACACACCAGCAGGTTATCA-3′, IL-10; (Forward) 5′-GGACAACATACTGCTAACCGACTC-3′ (Reverse) 5′- AAAATCACTCTTCACCTGCTCCAC-3′ (Supplementary Table 1).

#### Measurement of Cytokine Production

The effects of the EFEL6901 strain on the production of cytokines were determined in LPS-induced mouse peritoneal macrophage cells using an enzyme-linked immunosorbent assay (ELISA) kit Kang et al. (2016) with slight modification. Male BALB/c mice aged 7 weeks were purchased from Koatech (PyungTek, Korea) and underwent 1 week of adjustment prior to the experiments. For macrophage isolation, mice were injected intraperitoneally with 2 mL of 3% sterile thioglycollate medium (BD, Sparks, MD, United States). Three days later, the mice were sacrificed, and macrophages were collected by peritoneal lavage with DMEM (Hyclone). After centrifugation at 1500 × *g* for 10 min, cells were suspended in DMEM with 10% FBS and 1% penicillin-streptomycin and incubated overnight in a humidified atmosphere of 5% CO_2_ at 37°C. Non-adherent cells were removed for further analyses. Macrophages at 4 × 10^5^ cells/mL were stimulated with LPS (100 ng/mL) with or without heat-killed strains for 24 h. After incubation, the supernatant from each well was collected and the IL-10 and IL-12 secreted into the culture media were quantified using ELISA assay kits (R&D systems Mouse DuoSet ELISA IL-10 and IL-12, Minneapolis, MN, United States).

### *In vivo* Animal Test

#### Induction of Colitis in Mice by DSS

Female BALB/c mice aged 15 weeks were randomly divided into three groups of seven mice each, per the following experimental conditions: control mice with the administration of PBS (control group); DSS-treated mice (DSS group); DSS-treated mice with EFEL6901 (DSS + EFEL6901 group). The detailed experimental design as proposed by Rodríguez-Nogales et al. (2018) with slight modifications is shown in Supplementary Figure 1. Mice were orally administered EFEL6901 (1 × 10^9^ CFU suspended in 200 μL of PBS), while the control and DSS groups were treated with 200 μL of PBS for seven consecutive days. Subsequently, acute colitis was induced by adding 3% DSS (molecular mass 36,000–50,000 Da, 16011010; MP Biomedical, United States) to the drinking water for another 7 days. For the preparation of bacterial cells, the EFEL6901 strain was cultured in MRS broth (BD Difco) at 37°C for 24 h. Bacterial cells were harvested by centrifugation at 10,000 × *g* for 5 min, washed twice with PBS, and resuspended in PBS to a concentration of 1 × 10^9^ CFU/200 μL. Fresh bacteria were prepared daily in this way before administration to mice. The body weight of the mice was measured, and the percentage weight change relative to the initial weight prior to day 0 was calculated. The disease activity index (DAI) was calculated using the percent of weight loss, stool consistency, and presence of blood in the feces (Supplementary Table 2), as described by Jang et al. (2019). After 15 days, the mice were sacrificed, and colon lengths were measured. The animal protocol was approved (KHGASP-20-134) by the Institutional Animal Care and Use Committee of Kyung Hee University, and mice were cared for according to the specifications of the United States National Research Council for the Care and Use of Laboratory Animals (1996).

#### Measurement of Histological Score

The distal colon samples were washed with PBS, fixed in 10% formaldehyde, and stained with hematoxylin and eosin (H&E, Sigma). The histological score of each mouse was calculated according to cell infiltration and loss of crypt architecture, as proposed in a previous study (Wirtz et al., 2017). The specimens were analyzed blindly by a pathologist under a light microscope, and the scores were recorded.

#### Measurement of Expression Levels of Tight Junction Proteins

The expression levels of tight junction proteins such as E-cadherin, occludin, and claudin-3 were measured by western blot analysis (Jang et al., 2019). The colon samples were homogenized in 1× RIPA buffer (50 mM Tris–HCl, pH 7.5, 150 mM NaCl, 1 mM EDTA, 20 mM NaF, 0.5% NP-40, and 1% Triton X-100) with a protease inhibitor cocktail (Quartett, Berlin, Germany) using TissueLyser2 (Qiagen, Hilden, Germany). After centrifugation at 13,000 × *g* for 10 min, the supernatant was collected and the protein concentration was measured by Bradford assay (Thermo Fisher Scientific, Waltham, MA, United States) using albumin as a standard. An equal amount of protein from each sample was separated on 8∼10% sodium dodecyl sulfate polyacrylamide gel (SDS-PAGE) at 120 V for 60 min, and separated proteins were transferred to PVDF membrane (Bio-RAD, 1620177). The membrane was blocked with 5% skim milk in Tris-buffered saline and Tween 20 (TBS-T) for 1 h and incubated with a primary antibody against E-cadherin (Cell Signaling Technology, 3195S, Danvers, MA, United States), occludin (Thermo Fisher Scientific, 40-4700), claudin-3 (Thermo Fisher Scientific, 34-1700), and β-actin (Cell Signaling Technology, 4970S) overnight at 4°C. The membrane was washed three times with TBS-T and incubated with the secondary anti-rabbit horseradish peroxidase-conjugated antibody (diluted 1:5000 in 5% skim milk in TBS-T) for 1 h. The protein bands were detected with EzWestLumi plus (ATTO, Tokyo, Japan) and analyzed using an EZ-Capture MG (ATTO).

#### Measurement of Cytokine mRNA Expression Levels

Total RNA was extracted from each colon sample using TRIzol Reagent (Invitrogen) according to the manufacturer’s protocol. Because DSS inhibits the activities of both reverse transcriptase and polymerase, RNA was purified to remove DSS from colonic RNA using lithium chloride purification as described by Viennois et al. (2013). Measurement of mRNA expression levels was performed as described in section “Measurement of **N**itric **O**xide Synthase (iNOS) and Cyclooxygenase-2 (COX-2) mRNA Expression Level.” The results were analyzed after normalization with GAPDH as the reference gene. Relative expression levels of target genes were calculated using the ΔΔCt method. The specific primer sequences used are listed in section “Measurement of Nitric Oxide Synthase (iNOS) and Cyclooxygenase-2 (COX-2) mRNA Expression Level” (Supplementary Table 1).

#### Measurement of Short Chain Fatty Acids Levels

The levels of short chain fatty acids (SCFAs) in cecal contents were analyzed by high performance liquid chromatography (HPLC) (Liu et al., 2019). The intact cecum (90 mg) was suspended in deionized water (1 mL), homogenized for 1 min, and centrifuged at 13,000 × *g* for 5 min at 4°C. The supernatant was filtered through a 0.22-μm PVDF filter, and 20 μL was injected for HPLC analysis. Lactate, acetate, propionate, and butyrate concentrations were determined using a 1260 Infinity HPLC (Agilent, Santa Clara, CA, United States) equipped with an Aminex HPX-87H column (300 × 7.8 mm, Bio-Rad) and UV detector (Agilent) at a wavelength of 215 nm. Degassed 0.008 N H_2_SO_4_ was used as the eluent at a flow rate of 0.6 mL/min at room temperature.

### Microbiome Analysis

Total genomic DNA was extracted from three cecum samples randomly selected from each group using the QIAamp PowerFecal Pro DNA Kit (Qiagen). The V3-V4 region of the 16S rRNA gene was amplified using a primer set of 341-F (5′-CCTACGGGNGGCWGCAG-3′) and 785-R (5′-GACTACHVGGGTATCTAATCC-3′) (Klindworth et al., 2013). The 16S rRNA gene library was prepared using the Nextera Index Kit (Illumina, San Diego, CA, United States). The final PCR amplicon was sequenced by Macrogen (Seoul, Korea) using a MiSeq sequencer (Illumina). Raw FASTQ files were demultiplexed using QIIME2 based on their unique barcodes. Illumina FASTQ files for experiments are available from National Centre for Biotechnology Information (NCBI) BioProject PRJNA758218 under the following SRA (sequence read archive) accession numbers: control; SRX11945690, SRX11945691, and SRX11945692, colitis; SRX11945693, SRX11945694, and SRX11945695, colitis with EFEL6901; SRX11945696, SRX11945697, and SRX11945698.

### Starter Property During Kimchi Fermentation

#### Preparation of Simulated Kimchi Juice

Simulated kimchi juice (SKJ) was prepared with 700 g of cabbage, 200 g of radish, 50 g of leek, 10 g of ginger, 20 g of garlic, 3% (w/w) of salt, and 0.5% of fish peptone (Lee et al., 2017). Raw materials (cabbage, radish, garlic, ginger, and leek) were purchased from a local market. All of the materials were finely chopped using a physical blender, salt was added to the mixture, and kept overnight. Fish peptone (Bision, Seongnam, Korea) was added to the kimchi juice instead of the more traditional jeotgal. The SKJ was pasteurized at 70°C for 30 min. After cooling to room temperature, the mixture was centrifuged at 7,000 × *g* for 10 min to remove the pulp, and only the supernatant was used in the experiments.

#### Preparation of Nabak-Kimchi

Nabak-kimchi is a beverage-like kimchi mainly made of sliced cabbage and radish, and it was prepared according to a Korean commercial kimchi manufacturing method from the Daesang FNF (Icheon, Korea). Briefly, the vegetables and seasoning ingredients were washed, and then mixed using the following weight ratio; cabbage: radish: red pepper powder: red pepper: garlic: ginger: leek: water celery: carrot: pear: starter: 1.8% (w/v) salted water = 18: 12: 2: 1: 0.5: 0.3: 1: 1: 1: 0.5: 1: 61.7. Three paired sets of nabak-kimchi were inoculated with 1% (v/v) starter (*Le. mesenteroides* DRC1506 or *L. reuteri* EFEL6901) to a concentration of 10^6^ CFU/mL. The prepared nabak-kimchi were incubated at 10°C until optimal acidity states.

#### Microbial Growth Rate in SKJ

The bacterial strains were cultured in SKJ at 37°C and 30°C for 24 h. The optical density at 600 nm was measured using a spectrophotometer (BioTek), and the pH value was measured using an pH meter (Orion Star A211, Thermo Fisher Scientific).

#### Analysis of Fermentation Profiles in Nabak-Kimchi

Fermentation profiles in the starter-inoculated nabak-kimchi were determined by measuring pH, total titratable acidity (TTA), sugars, and organic acids (Yu et al., 2019). The pH of nabak-kimchi was measured using a pH meter (Orion Star A211, Thermo Fisher Scientific.). TTA was measured by 0.01 N NaOH and was calculated as percent of lactic acid within the samples. Contents of sugars and organic acids were measured using ^1^H-NMR spectroscopy. Briefly, 3 mL of each of kimchi supernatants were adjusted to pH 6.0, and resuspended in 3 mL of 99.9% deuterium oxide (D_2_O; Sigma) containing 1 mM sodium 2,2-dimethyl-2-silapentane-5-sulfonate (DSS; Sigma). The suspended mixtures were centrifuged at 13,000 × *g* for 5 min and 750 μL of the supernatants were transferred into a 5 mm NMR tube. Spectra were acquired using a Bruker 500-MHz NMR spectrometer (Bruker Magnetics., Fällanden, Switzerland) at 25°C. Identification and quantification of individual metabolites from the ^1^H-NMR spectra were performed using the Profiler module of the Chenomx NMR Suite program (ver. 6.1; Chenomx, Canada).

### Statistical Analysis

All data except *in vivo* results are presented as mean ± standard deviation (SD). Data from the *in vivo* test are expressed as the mean ± standard error of the mean (SEM). Statistical analysis was performed using IBM SPSS software version 22 (SPSS Inc., Chicago, IL, United States). The independent *t*-test was used to analyze differences between two groups, and one-way analysis of variance (ANOVA) was used to analyze differences between multiple groups using Duncan’s method. Principal component analysis (PCA) was performed using R statistical software (R Core Team, Vienna, Austria).

## Results

### Safety and Stability of EFEL6901

**FIGURE 1:**
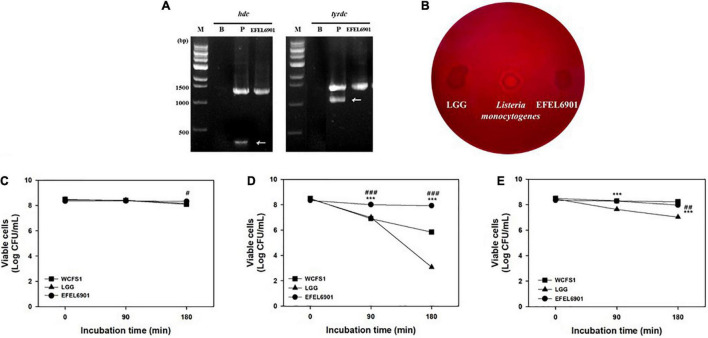
Safety and stability assessment of *Limosilactobacillus reuteri* EFEL6901 strains. **(A)** Detection of genes related to biogenic amine production. Lane M, 1 kb DNA marker; lane 1, negative control which has no template DNA; lane 2, positive controls having *hdc* (histidine decarboxylase, 440 bp), and *tyrdc* (tyrosine decarboxylase, 1100 bp) genes from *L. reuteri* ATCC 23272 and *Enterococcus faecalis* KCCM 11729, respectively. **(B)** Hemolytic activity analysis of *L. reuteri* EFEL6901. Hemolytic activity was measured in BHI broth containing 7% horse blood. Left, the negative control, *L. rhamnosus* GG; center, positive control, *Listeria monocytogenes*; right, the EFEL6901 strain, showing clear zone around the cell drop. **(C)** Viability of *L. reuteri* EFEL6901 at pH 3.0. **(D)** Viability of *L. reuteri* EFEL6901 at pH 2.5. **(E)** Viability of *L. reuteri* EFEL6901 in 0.3% bile salt. Significant differences are presented with *L. plantarum* WCFS1 (^#^*p* < 0.05, ^##^*p* < 0.01, and ^###^*p* < 0.001) or *L. rhamnosus* GG (*p < 0.05, **p < 0.01, and ***p < 0.001).

To assess the safety of the EFEL6901 strain, the presence of genes related to biogenic amine production was examined by multiplex PCR. As shown in Figure 1A, the EFEL6901 strain did not exhibit the presence of *hdc* and *tyrdc* genes, which are responsible for the production of histamine and tyramine, respectively. Meanwhile, *hdc* and *tyrdc* genes were detected in the positive control strains, *L. reuteri* ATCC 23272 and *E. faecalis* KCCM 11729, respectively.

To evaluate the hemolytic activity of the EFEL6901 strain, bacterial cells were inoculated on horse blood agar medium and incubated at 37°C for 24 h. As shown in Figure 1B, the EFEL6901 strain did not exhibit a clear area around the cell drop, whereas the positive control, *L. monocytogenes* did exhibit a clear area, which could be interpreted as hemolysis activity. These results indicate that the EFEL6901 strain did not have biogenic amine genes or hemolytic activity, so the strain is safe for use in food fermentation.

To investigate the stability of EFEL6901 in the human GI tract, acid and bile salt tolerance levels were measured. As shown in Figures 1C,D, the viability of EFEL6901 was maintained at pH 3.0, and pH 2.5, whereas LGG and WCFS1, which were used as positive controls, significantly decreased at pH 2.5. The results of the 0.3% bile salt tolerance test (Figure 1E) were that EFEL6901 showed higher viability compared to LGG, but lower than that of WCFS1.

These results indicate that the EFEL6901 strain is safe for humans and approximately as stable in the GI environment as LGG and WCFS1, which are known to be resistant to acid and bile salts.

### Adhesion and Antioxidative Activities of EFEL6901

**FIGURE 2:**
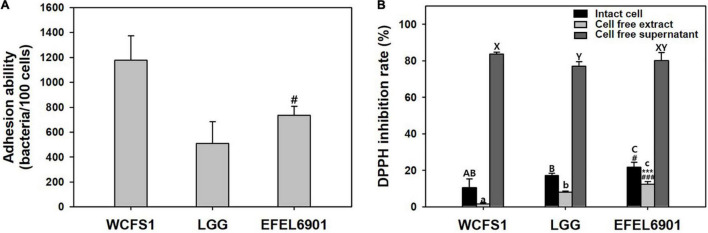
Adhesion ability **(A)** and antioxidative activity **(B)** of *Limosilactobacillus reuteri* EFEL6901. **(A)** Intestinal adhesion ability of *L. reuteri* EFEL6901 on HT-29 cells was measured. **(B)** Antioxidative activity was measured by DPPH inhibition assay. Significant differences are presented with *L. plantarum* WCFS1 (^#^*p* < 0.05, ^###^*p* < 0.001) or *L. rhamnosus* GG (***p < 0.01).

The ability of EFEL6901 to adhere to intestinal epithelial cells was measured by incubating bacterial cells with HT-29 cells (MOI 100:1). As shown in Figure 2A, WCFS1 and LGG adhered to HT-29 cells at 1,178 CFU and 509 CFU per 100 cells, respectively. EFEL6901 showed the same high adhesion ability at 736 CFU per 100 cells, comparable to LGG.

The scavenging of DPPH free radicals is attributed to the hydrogen-donating ability of antioxidants and is used for antioxidant assays. Intact cells, cell-free extracts, and cell-free supernatant of EFEL6901 were prepared, and their antioxidative activities were measured (Figure 2B). Intact EFEL6901 cells showed a 21.6% DPPH inhibition rate, which was significantly higher than that of WCFS1 (10.6%) or LGG (17.1%). The cell-free extract of EFEL6901 showed a 12.4% DPPH inhibition rate, which was also significantly higher than that of WCFS1 (1.6%) and LGG (8.0%). In addition, the cell-free supernatant showed an 80.2% DPPH inhibition rate, which was significantly higher than LGG (77.1%) but lower than that of WCFS1 (83.7%). Therefore, EFEL6901 is considered to have excellent antioxidant activity in both intact cells and cell-free extracts.

### Anti-inflammatory Activity of EFEL6901

**FIGURE 3:**
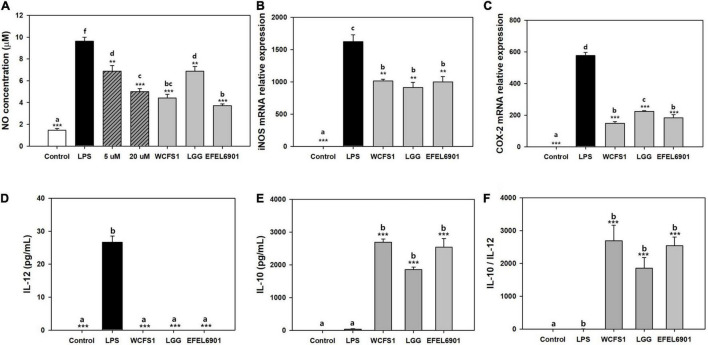
Anti-inflammatory effect of *Limosilactobacillus reuteri* EFEL6901 in LPS-induced RAW 264.7 cells **(A–C)** and LPS-induced mouse peritoneal macrophage cells **(D–F)**. Effects of heat-killed *L. reuteri* EFEL6901 on the production of NO **(A)**, mRNA expression of iNOS **(B)** and COX-2 **(C)** in LPS-induced RAW 264.7 cells were measured after 24 h. Expressed mRNA levels of iNOS and COX-2 were determined by real-time PCR. Effects of heat-killed *Lactobacillus reuteri* EFEL6901 strain on IL-12 **(D)**, IL-10 **(E)** and the calculated ratio IL-10/IL-12 **(F)** in LPS-induced mouse peritoneal macrophages. Cells were treated with heat-killed bacteria and stimulated with LPS for 24 h. The levels of IL-12 and IL-10 in cell culture supernatants were measured by ELISA kits. Different letters indicate a significant difference at *p* < 0.05 according to Duncan’s multiple range test. Significant differences are presented with LPS positive group: *p < 0.05; **p < 0.01; ***p < 0.001.

To determine the anti-inflammatory activity, NO production of the heat-killed EFEL6901 strain was measured in LPS-induced RAW 264.7 cells (Figure 3). As shown in Figure 3A, treatment with LPS significantly increased the production of NO compared to the control (*p* < 0.001). However, treatment with methyl arginine, which is a NO synthase inhibitor, suppressed NO production in a dose-dependent manner (*p* < 0.01, *p* < 0.001). Similarly, all heat-killed bacterial cells exhibited significant inhibitory activity against NO production. In particular, EFEL6901 showed significant inhibitory activity compared to that of other commercial probiotics. This result indicates that EFEL6901 cells have an anti-inflammatory capacity that affects immune cells in the human GI tract.

Next, RT-qPCR was performed to determine whether the inhibitory effect of the EFEL6901 strain on NO production was associated with the transcription levels of iNOS (Figure 3B) and COX-2 (Figure 3C) genes in RAW 264.7 cells. Treatment of RAW 264.7 cells increased the mRNA expression levels of iNOS and COX-2, whereas heat-killed EFEL6901 significantly inhibited the expression levels of iNOS (Figure 3B, *p* < 0.01) and COX-2 (Figure 3C, *p* < 0.001), similar to WCFS1 and LGG. This result indicates that treatment with heat-killed EFEL6901 inhibits NO production by inhibiting the expression of iNOS and COX-2 in RAW 264.7 cells.

To investigate the effects of EFEL6901 on cytokine production in LPS-induced mouse peritoneal macrophages, cells were treated with heat-killed bacteria for 24 h, and the production of pro-inflammatory cytokine IL-12 (Figure 3D) and anti-inflammatory cytokine IL-10 (Figure 3E) were analyzed. LPS treatment markedly increased the production of IL-12, but not that of IL-10. Meanwhile, treatment with heat-killed EFEL6901 completely inhibited IL-12 and significantly induced IL-10 release, leading to a high anti-inflammatory index, the IL-10/IL-12 ratio (Figure 3F), similar to those of WCFS1 and LGG. In summary, EFEL6901 cells were potent inducers of IL-10 and inhibitors of IL-12, exerting strong anti-inflammatory activity in both *in vitro* and *ex vivo* tests.

### *In vivo* Animal Test

#### Inflammatory Symptoms and Histological Score of DSS-Induced Colitis Mice

**FIGURE 4:**
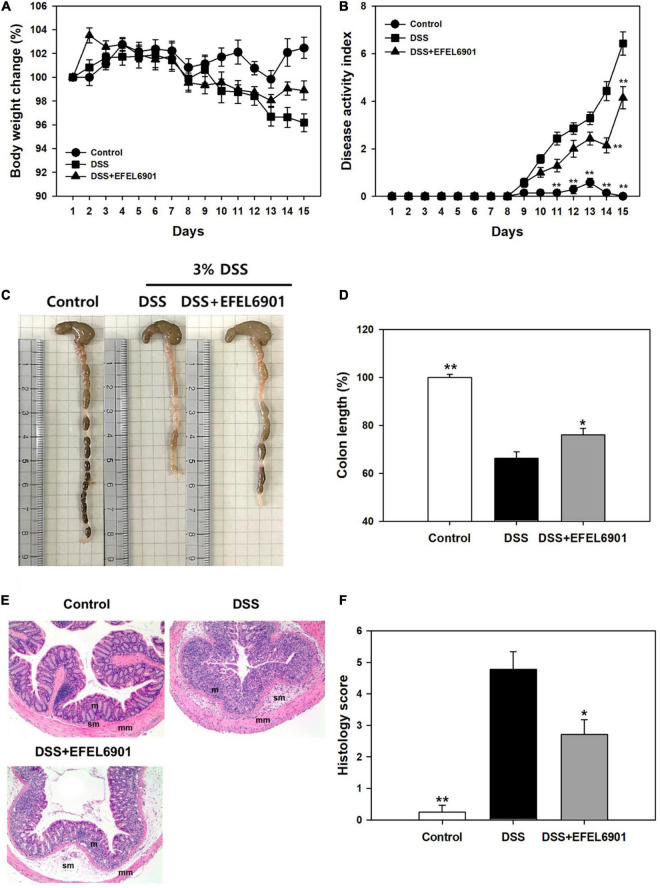
Effects of *Limosilactobacillus reuteri* EFEL6901 strain on improvement of symptoms in DSS-induced murine colitis model. The body weight **(A)** and the disease activity index score **(B)** were evaluated throughout the experiment. After 15 days, the mice were sacrificed and colon lengths **(C,D)** were measured. **(E)** Histological features of a representative colon of each group. m, mucosa; sm, submucosa; mm, muscular layer. Mag. = X100 for all. **(F)** The histological score combining inflammatory cell infiltration and loss of intestinal glands. Results are expressed as means ± standard error (*n* = 7). Significant differences are presented with the DSS group: *p < 0.05; **p < 0.01.

The anti-inflammatory effects of the EFEL6901 strain were evaluated using a dextran sulfate sodium (DSS)-induced colitis mouse model, as shown in Supplementary Figure 1. Briefly, a group of mice was orally administered EFEL6901 for 7 days, while the control and DSS groups were treated with PBS. Acute colitis was then induced using 3% DSS solution in the DSS group and DSS + EFEL6901 group for another 7 days. As shown in Figure 4A, the body weight of mice in the DSS group was significantly decreased from day 11 (*p* < 0.05) compared with the control group, while the body weight loss in the DSS + EFEL6901 group was significantly reduced on day 15 (*p* < 0.05).

To evaluate the severity of inflammatory symptoms, the disease activity index (DAI) was calculated using the percent weight loss, stool consistency, and presence of blood in the feces, as described in Supplementary Table 2. The DAI score of the DSS group increased to 6.43 ± 0.49 on day 15, whereas the score of DSS + EFEL6901 group reached to 4.14 ± 0.47, which was significantly lower than that of the DSS group (Figure 4B).

When the colon length of the mice groups was measured (Figures 4C,D), the length of those from the DSS group (5.7 ± 0.2 cm) was significantly shorter than from of the control group (8.6 ± 0.1 cm) (*p* < 0.01). Meanwhile, the DSS + EFEL6901 group had significantly longer colon length (6.6 ± 0.2 cm) than the DSS group (*p* < 0.01). These results revealed that intestinal inflammation was effectively prevented by the introduction of EFEL6901.

Additionally, to analyze the severity of colonic inflammation, hematoxylin and eosin (H&E) staining was conducted on the distal colon (Figure 4E). Increased mucosal damage and infiltration of inflammatory cells into the mucosa occurred in the DSS group compared to the control group. In contrast, DSS + EFEL6901-treated mice exhibited less inflammatory cell infiltration in the mucosal tissue and an intact colonic architecture with less apparent ulceration. The histological score of DSS + EFEL6901-treated mice (2.71 ± 0.47) was significantly lower (*p* < 0.05) than that of DSS-treated mice (4.77 ± 0.56) (Figure 4F). These results support the conclusion that EFEL6901 treatment can alleviate the symptoms of murine colitis induced by DSS.

#### Tight Junction Proteins

**FIGURE 5:**
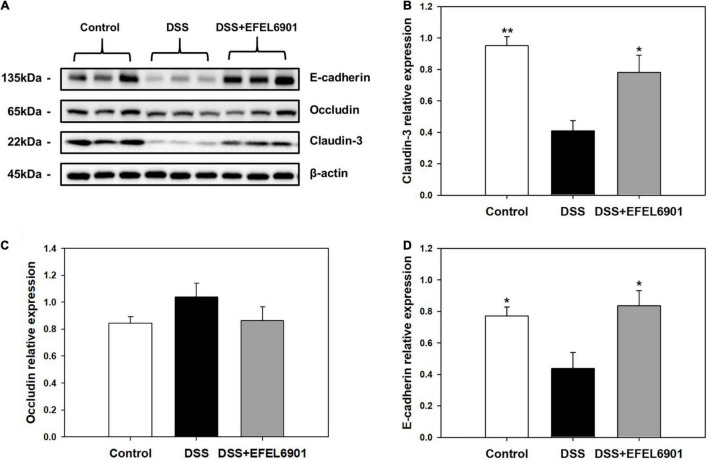
Effects of *Limosilactobacillus reuteri* EFEL6901 strain on tight junction protein levels in mouse colon. **(A)** Representative western blotting of E-cadherin, occludin, claudin-3, and β-actin (loading control). Quantification of E-cadherin **(B)**, occludin **(C)**, and claudin-3 **(D)** protein expression levels in the colon. Results are expressed as means ± standard error (*n* = 7). Significant differences are presented with the DSS group: *p < 0.05; **p < 0.01.

Tight junction proteins such as E-cadherin, occludin, and claudin-3 are involved in barrier function in the intestine, and their low expression levels are closely related with DSS-induced colitis (Eichele and Kharbanda, 2017). As shown in Figure 5, the expression levels of tight junction proteins in the mouse colon were measured by western blotting. The expression of E-cadherin and claudin-3 was decreased in the DSS group compared to that in the control group (*p* < 0.05, *p* < 0.01), whereas their expression levels in the DSS + EFEL6901 group were significantly higher than those in the DSS group. This result indicates that EFEL6901 has the capacity to restore the epithelial barrier disruption induced by DSS.

#### Cytokine mRNA Expression Levels

**FIGURE 6:**
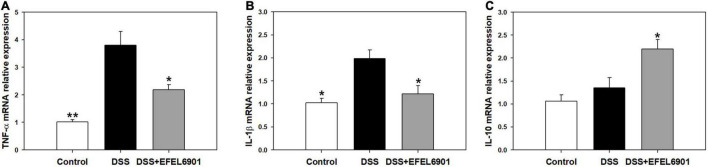
Effects of *Limosilactobacillus reuteri* EFEL6901 strain on cytokine levels in mouse colon. Quantification of TNF-α **(A)**, IL-1β **(B)**, and IL-10 **(C)** mRNA expression levels in the colon. Expressed mRNA levels of cytokines were determined by real-time PCR. Results are expressed as means ± standard (*n* = 5). Significant differences are presented with the DSS group: *p < 0.05; **p < 0.01.

Cytokines are involved in the etiology of IBD by regulating intestinal mucosal inflammation and epithelial integrity (Egger et al., 2000). To compare the expression levels of cytokines related to inflammation in mice groups, the mRNA levels of pro-inflammatory cytokines, TNF-α and IL-1β, as well as anti-inflammatory IL-10, were measured by RT-qPCR. As shown in Figure 6, the mRNA expression levels of TNF-α and IL-1β were significantly increased (*p* < 0.05, *p* < 0.01) in the DSS group compared with the control group, whereas the level of IL-10 was significantly increased (*p* < 0.05) in the DSS + EFEL6901 group. In conclusion, EFEL6901 downregulated pro-inflammatory cytokine genes and upregulated the expression of anti-inflammatory cytokine genes. This result indicates that that the observed therapeutic effect is associated with modulation of pro and anti-inflammatory proteins in the colon.

#### Microbiome Analysis

**FIGURE 7:**
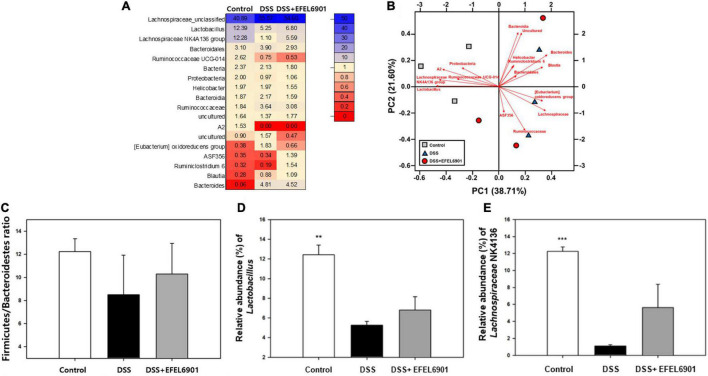
Bacterial taxonomic compositions in DSS-induced colitis mice after oral administration of *Limosilactobacillus reuteri* EFEL6901. **(A)** The differences heatmap of microbial abundance at genus level. **(B)** Biplot of the principal components analysis (PCA) of microbial abundance at genus level. **(C)** Relative abundance of Firmicutes and Bacteroidetes at the phylum level. **(D)** Relative abundance of *Lactobacillus* at the genus levels. **(E)** Relative abundance of *Lachnospiraceae* NK4A136 at the genus levels. Significant differences are presented with the DSS group: *p < 0.05; **p < 0.01.

Total genomic DNA was extracted from the contents of the cecum, and the diversity of the intestinal microbiota was analyzed by 16S rRNA amplicon sequencing. The gut microbiota composition with a high relative abundance at the phylum level mainly included Firmicutes and Bacteroidetes (Supplementary Figure 3A). The abundance of the two phyla accounted for more than 90% of the bacteria in the cecum. Compared with the control group, the relative abundance of Bacteroidetes increased in the DSS group, whereas its abundance tended to decrease after administration of EFEL6901. Notably, the Firmicutes/Bacteroidetes (F/B) ratio was lower in the DSS group (8.51) than in the control group (12.24), and the F/B ratio in the DSS + EFEL6901 group slightly increased to 10.30, but this result was not significant (*p* > 0.05) (Figure 7C).

At the genus level, *Lachnospiraceae_unclassified* and *Lactobacillus* spp. were the most prevalent genera, followed by the *Lachnospiraceae* NK4A136 group (Figures 7A,B). The relative abundance of beneficial flora such as *Lactobacillus* and *Lachnospiraceae* NK4A136 group in control mice was significantly higher than in DSS mice (*p* < 0.01, *p* < 0.001) (Figures 7D,E). In contrast, *Lactobacillus* and *Lachnospiraceae* NK4A136 group tended to slightly increase after oral administration of EFEL6901, but this result was not significant (*p* > 0.05). Taken together, these results indicate that administration of EFEL6901 can partially restore the microbial dysbiosis induced by DSS in mice (Figure 7B).

#### Short-Chain Fatty Acid Levels

**FIGURE 8:**
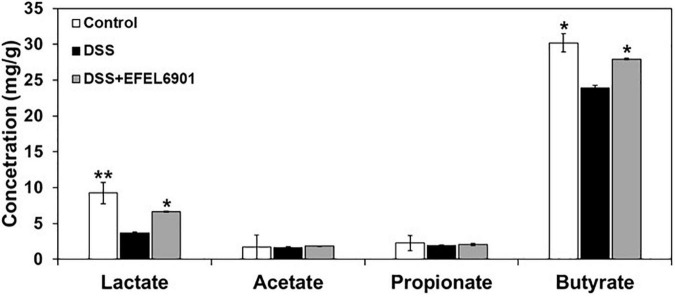
Effects of *Limosilactobacillus reuteri* EFEL6901 strain on short-chain fatty acids (SCFAs) levels in mouse cecum. Significant differences are presented with the DSS group: *p < 0.05; **p < 0.01.

Short-chain fatty acids (SCFAs) such as lactate, acetate, propionate, and butyrate are mainly produced by beneficial microorganisms in the intestine, and they help to maintain intestinal homeostasis and alleviate colitis (Liu et al., 2019). To compare their profiles with microbial diversity in each mouse group, their concentrations were measured in the cecum compositions (Figure 8). In the DSS group, the concentrations of lactate and butyrate were significantly lower than those in the control group (*p* < 0.05, *p* < 0.01), whereas their levels in the DSS + EFEL6901 group were comparable to those in the control group. This result indicates that the decrease in SCFAs was caused by DSS and could potentially be restored by EFEL6901 administration.

### Starter Property During Kimchi Fermentation

**FIGURE 9:**
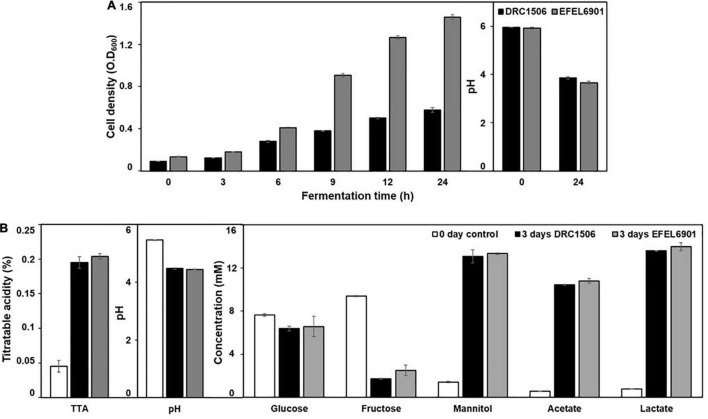
Fermentation profiles of *Limosilactobacillus reuteri* EFEL6901 in simulated kimchi juice (SKJ) **(A)** and nabak-kimchi **(B)** showing changes of cell density, titratable acidity, pH, carbon sources (glucose and fructose), and metabolites (mannitol, acetate, and lactate). **(A)** *L. reuteri* EFEL6901 and *Leuconostoc mesenteroides* DRC1506, a commercial kimchi starter, were cultured in SKJ at 37°C and 30°C, respectively. **(B)** *L. reuteri* EFEL6901 and *Le. mesenteroides* DRC1506 were inoculated in nabak-kimchi and incubated at 10°C for 3 days.

To investigate the suitability of EFEL6901 as a starter for kimchi fermentation, its properties were tested in two different kimchi conditions, SKJ and nabak-kimchi (Figure 9). Firstly, to test the adaptability of the strain in kimchi environment containing various antimicrobial compounds and polyphenols, the growth rates of the strain and the commercial starter, *Le. mesenteroides* DRC1506, were measured at their optimal temperatures, 37°C and 30°C, respectively (Figure 9A). As a result, EFEL6901 showed fast growth, reaching up to OD_600nm_ = 1.35 after 24 h, higher than DRC1506 (OD_600nm_ = 0.45), and the pHs of two fermented juices decreased to 3.9. The result indicates that EFEL6901 is a well-adapted lactic acid bacterium in the kimchi condition. Additionally, to investigate the fermentation property of EFEL6901 in kimchi, the two strains, EFEL6901 and DRC1506, were individually inoculated in nabak-kimchi followed by incubation at 10? until the pH reached the optimal ripening condition, and changes of pH, TTA, and metabolites of nabak-kimchi were analyzed (Figure 9B). As results, after 3 days of incubation, the pH and TTA of the EFEL6901-inoculated nabak-kimchi reached to 3.91 and 0.20%, respectively, and the levels were not significantly different from those of DRC1506-kimchi. Concentrations of glucose and fructose that are major free sugars in kimchi decreased to 6.58 ± 0.94 mM and 2.51 ± 0.49 mM, respectively. Meanwhile, concentrations of acetate (10.81 ± 0.22 mM), lactate (13.99 ± 0.36 mM), and mannitol (13.34 ± 0.08 mM) increased along with the fermentation. When compared the metabolite concentrations synthesized during the period, there were no differences between two kimchi samples, revealing similar fermentation property of EFEL6901 with the commercial strain. These results demonstrate that EFEL6901 is well-adapted to kimchi condition to synthesize the major metabolic compounds affecting kimchi taste.

## Discussion

Recently, researchers have studied the use of probiotic starters to improve the health-promoting effects of fermented foods (Bonatsou et al., 2017; Roobab et al., 2020). For instance, a starter culture for cheese, *Propionibacterium freudenreichii*, exhibited an immunomodulatory activity by preventing the induction of inflammatory markers in colitis cells (Plé et al., 2015; Rabah et al., 2018), and its surface layer proteins (SLPs) were responsible for the activity (do Carmo et al., 2017, 2018, 2019). Similarly, health-promoting probiotics can be delivered via fermented foods (Tamang et al., 2020; Sauer and Han, 2021).

*Limosilactobacillus reuteri* is gram-positive, facultative anaerobic, and heterofermentative lactic acid bacterium and it is frequently found in fermented vegetables, milk products, and the gastrointestinal tract of humans and other animals (Mauro et al., 2016; Mu et al., 2018). It has been known that *L. reuteri* is unique in ability to produce antibacterial molecule, reuterin, that is capable of inhibiting a wide spectrum of microorganisms (Chen and Chen, 2013). Recent researches have revealed its significant associations regarding obesity, atopic dermatitis, diarrhea, pathogen infection, chronic diseases, and modulation of gut microbiome (Mu et al., 2018; Wang et al., 2020). In addition, several studies have reported that *L*. *reuteri* was a good starter culture for fermented milk without negative effects on the flavor and partially used for plant-based fermentations such as pineapple, pumpkin, and carrot juices (Mauro et al., 2016; Ortiz-Rivera et al., 2017; Bintsis, 2018). In this study, we developed an anti-inflammatory probiotic starter for kimchi fermentation, as kimchi is considered a popular food matrix to deliver probiotics with daily meals.

The EFEL6901 strain was safe for use in foods and was stable under human gastrointestinal conditions. Amino acid decarboxylase genes (*hdc*, *tyrdc*) and hemolytic activity were not found in EFEL6901. Acidic conditions in the gastrointestinal tract can oxidize and destroy important biomolecular compounds, and bile salts in the body cause disorganization of cell membranes, resulting in cell death due to leakage of cell contents (Sahadeva et al., 2011; Kim et al., 2019). EFEL6901 showed higher resistance to acid than did the two positive control strains and showed higher bile salt tolerance than LGG. In addition, probiotics that have survived in the stomach and small intestine should be able to colonize properly in the colon after adhesion to colonic epithelial cells. Adhesive ability to the colon not only promotes immunoregulatory effects, but also stimulates gut barrier and metabolic functions, and serves as a protection against enteropathogens through a variety of mechanisms, including competition for host cell binding sites (Monteagudo-Mera et al., 2019). EFEL6901 showed similar adhesion ability to Caco-2 and HT-29 compared to LGG, which is known to have high adhesion ability.

The EFEL6901 strain exhibited antioxidant and anti-inflammatory activities as a health-promoting probiotic. Antioxidant activity is regarded as an important characteristic of probiotics because oxidation causes several diseases, such as non-alcoholic fatty liver disease, age-related diseases, and cancer (Zhang et al., 2013). The antioxidant activities of lactic acid bacteria are attributed to the cell-surface compounds of intact cells, such as extracellular polysaccharides and lipoteichoic acid, and several lactic acid bacteria are known to secrete intracellular antioxidant enzymes (Xing et al., 2015). Consistent with the literature, EFEL6091 exhibited strong antioxidative activity in both intact cells and cell-free extracts. Large amounts of NO are produced by iNOS in immune cells when stimulated by inflammatory inducers, such as LPS and several pro-inflammatory cytokines (Salim et al., 2016). Our results showed that treatment with heat-killed EFEL6901 decreased NO production via downregulation of iNOS in LPS-induced RAW 264.7 cells, whereas lysate fraction did not (data not shown), which is consistent with a previous report using *L. fermentum* (Rodríguez-Nogales et al., 2015). This result reveals that aberrantly activated macrophage was modulated due to binding of LPS with EFEL6901 cell surface layer proteins thereby inhibiting NO production from macrophage (Thomas and Versalovic, 2010). In addition, the EFEL6901 strain strongly induced the anti-inflammatory cytokine IL-10 and completely inhibited the pro-inflammatory cytokine IL-12, resulting in a high ratio of IL-10/IL-12 in LPS-induced mouse macrophage cells (Figure 3). The ratio of IL-10 and IL-12 is used to evaluate the anti-inflammatory properties of microorganisms in *in vitro* and *in vivo* colitis mouse models, and a high ratio of IL-10/IL-12 represents high anti-inflammatory activity (Foligne, 2007; Gad et al., 2011; Jo et al., 2020).

For the *in vivo* test, we used a DSS-induced colitis mouse model, which is a chemical induction method of intestinal inflammation similar to human ulcerative colitis, suitable for IBD studies because of its rapidity, simplicity, and reproducibility (Eichele and Kharbanda, 2017). DSS is a negatively charged sulfated polysaccharide that is water-soluble, and murine colitis is caused by the administration of DSS in water. The most well-known mechanism of DSS is causing damage to colon epithelial cells, which increases the entry of lumenal bacteria and immune cells and allows the release of proinflammatory cytokines (Chassaing et al., 2014; Eichele and Kharbanda, 2017). High levels of pro-inflammatory cytokines, such as TNF-α, IL-1β, and IL-12, are associated with DSS-induced colitis in mice (Bauer et al., 2010). On the other hand, the anti−inflammatory cytokine IL−10 is secreted by multiple cell types—monocytes, T cells, and B cells—which play an important role in the maintenance of intestinal homeostasis (Iyer and Cheng, 2012). Therefore, the ability of probiotics to enhance the production of IL-10 and reduce pro-inflammatory cytokines is important for the treatment of IBD. In our study, EFEL6901 downregulated the expression of pro-inflammatory cytokines TNF-α and IL-1β, and upregulated the level of the colonic anti-inflammatory cytokine IL-10 (Figure 6). Similarly, the effects of downregulated pro-inflammatory cytokines via probiotics strains such as *L. reuteri*, *Bacillus coagulans*, *Bifidobacterium longum*, and *Clostridium butyricum* were investigated in a DSS-induced colitis mouse model (Wang et al., 2020). In addition, this result showed the similar inhibition effect of pro-inflammatory cytokine with LGG and WCSF1 which were used as positive controls *in vitro* test (Smelt et al., 2013; Yan et al., 2017).

Short-chain fatty acids produced by microorganisms are abundant in the intestinal tract and play a crucial role in maintaining intestinal homeostasis (Liu et al., 2019). In IBD patients and colitis animal models, decreased SCFAs have been frequently observed (den Besten et al., 2013; Liu et al., 2019). Among SCFAs, butyrate exerts anti-inflammatory effects by suppressing the activation of TNF-α-induced nuclear factor (NF-kB) (Venkatraman et al., 2003). Along with the previous results, it is possible to say that the increase of SCFAs, such as lactate and butyrate, could ameliorate colitis (Figure 8).

The Firmicutes/Bacteroidetes (F/B) ratio is associated with maintaining intestinal homeostasis, and changes in its value can lead to a variety of pathologies (Stojanov et al., 2020). For example, an increased F/B ratio is usually observed in obesity, and a decreased F/B ratio is observed in IBD. Several studies have observed that the F/B ratio decreases in a DSS-induced colitis mouse model (Dou et al., 2020) and in some Crohn’s disease and ulcerative colitis patients (Frank et al., 2007). Consistent with the previous results, our results showed that the F/B ratio was lower in the DSS group (8.51), medium in the DSS + EFEL6901 group (10.30), and high in the control group (12.24) (Figure 7). Anatomically, the mouse and human colon have prominent differences due to diet, feeding pattern, body size, and metabolic requirements (Treuting et al., 2011). Similarly, microbiota are slightly different between mice and humans; *Lactobacillus*, *Alistipes*, and *Turicibacter* are more abundant in the mouse gut, and *Prevotella*, *Faecalibacterium*, and *Ruminococcus* are more abundant in the human gut, whereas *Clostridium*, *Bacteroides*, and *Blautia* are similar in both (Nguyen et al., 2015). According to Håkansson et al. (2015) and Stadlbauer et al. (2020), the abundance of *Lactobacillus* and *Lachnospiraceae* NK4A136 groups was significantly reduced in DSS-induced colitis mice, which was consistent with our results (Figure 7 and Supplementary Figure 2). Both *Lactobacillus* and *Lachnospiraceae* NK4A136 are considered anti-inflammatory factors because of their ability to produce SCFAs. In particular, *Lachnospiraceae* NK4A136 is a potential probiotic that produces butyrate (Stadlbauer et al., 2020). The colitis mice showed a lower abundance of *Lactobacillus* and *Lachnospiraceae* NK4A136 groups than the control group (Dou et al., 2020), which is consistent with our results.

Our data suggest that EFEL6901 has therapeutic potential for treating IBD for multiple reasons. First, our study confirmed that EFEL6901 increased E-cadherin and claudin-3 levels, and that these changes in tight junction protein expression were considered a major factor in colitis relief. In addition, bacterial cell components of EFEL6901 trigger immunological responses to Toll-like and other signal transduction receptors in the intestinal epithelium, dendritic cells, and other immune intestinal cells. Indeed, EFEL6901 showed a significantly lower expression of pro-inflammatory cytokine genes and higher expression of anti-inflammatory cytokines than the DSS group. However, the molecular mechanism of this anti-inflammatory action is still unclear, and further research is needed.

Moreover, we evaluated the suitability of EFEL6901 as a kimchi starter. Factors involved in bacterial growth during kimchi fermentation include external conditions such as fermentation temperature and raw materials (cabbage, radish, garlic, red pepper powder, etc.) (Lee et al., 2020). Allicin derived from garlic is known to inhibit the growth of bacteria derived from various raw materials (Lee et al., 2020). It has also been reported that certain lactic acid bacteria that produce antibacterial substances can survive in garlic (Lim et al., 2015; Jo et al., 2020). EFEL6901 showed higher growth rates than DRC1506 in SKJ and at its optimum temperature, probably because it is resistant to the antibacterial activity of garlic and utilizes the nutritional components of kimchi (Figure 9A). Furthermore, another important characteristic of EFEL6901 is that it produced similar amounts of metabolites compared with those of DRC1506 in nabak-kimchi (Figure 9B).

This study has a few limitations. First, for safety assessment, we analyzed the presence of genes for biogenic amines and tested the hemolytic activity of *L. reuteri* EFEL6901. EFSA states that microorganisms in the Qualified Presumption of Safety list can be used as food additives; however, it is necessary to prove that they do not have a transmissible antibiotic resistance gene [EFSA Panel on Additives and Products or Substances used in Animal Feed (FEEDAP), 2012]. To meet the requirements of this guideline, further genome-based safety evaluation as well as antibiotic susceptibility tests should be performed. Secondly, to test the suitability of EFEL6901 as a starter for kimchi fermentation, we analyzed its fermentation property in nabak-kimchi and demonstrated its adaptability in kimchi condition to synthesize the major metabolic compounds affecting kimchi taste. Further studies are necessary to investigate the sensory characteristics of kimchi fermented by *L. reuteri* EFEL6901 by using GC-MS analysis and sensory test.

## Conclusion

In this study, we confirmed that *L. reuteri* EFEL6901 is safe and effective for use in food fermentation and has beneficial probiotic activities such as antioxidant properties, capacity to restore the gut epithelial barrier, and anti-inflammatory activity. Furthermore, *L. reuteri* EFEL6901 showed potential as a kimchi starter because it rapidly grew in SKJ and synthesize the major metabolic compounds affecting kimchi taste in nabak-kimchi.

## Data Availability Statement

The datasets presented in this study can be found in online repositories. The names of the repository/repositories and accession number(s) can be found below: https://www.ncbi.nlm. nih.gov/, PRJNA758218.

## Ethics Statement

The animal study was reviewed and approved by Institutional Animal Care and Use Committee of Kyung Hee University.

## Author Contributions

HeS, HK, and NH contributed to conception and design of the study. HeS, GK, YJ, SC, YS, and BR performed the study and statistical analysis. HeS and HyS wrote the first draft of the manuscript. All authors contributed to manuscript revision, read, and approved the submitted version.

## Conflict of Interest

BR was employed at the Daesang Corporation Research Institute. The remaining authors declare that the research was conducted in the absence of any commercial or financial relationships that could be construed as a potential conflict of interest.

## Publisher’s Note

All claims expressed in this article are solely those of the authors and do not necessarily represent those of their affiliated organizations, or those of the publisher, the editors and the reviewers. Any product that may be evaluated in this article, or claim that may be made by its manufacturer, is not guaranteed or endorsed by the publisher.
