# Supplementary figures and images for: Development of Anti-inflammatory Probiotic Limosilactobacillus reuteri EFEL6901 as Kimchi Starter: in vitro and In vivo Evidence
Source: Front Microbiol. 2021 Nov 25;12:760476. doi: 10.3389/fmicb.2021.760476 (PMC8656428; doi:10.3389/fmicb.2021.760476)

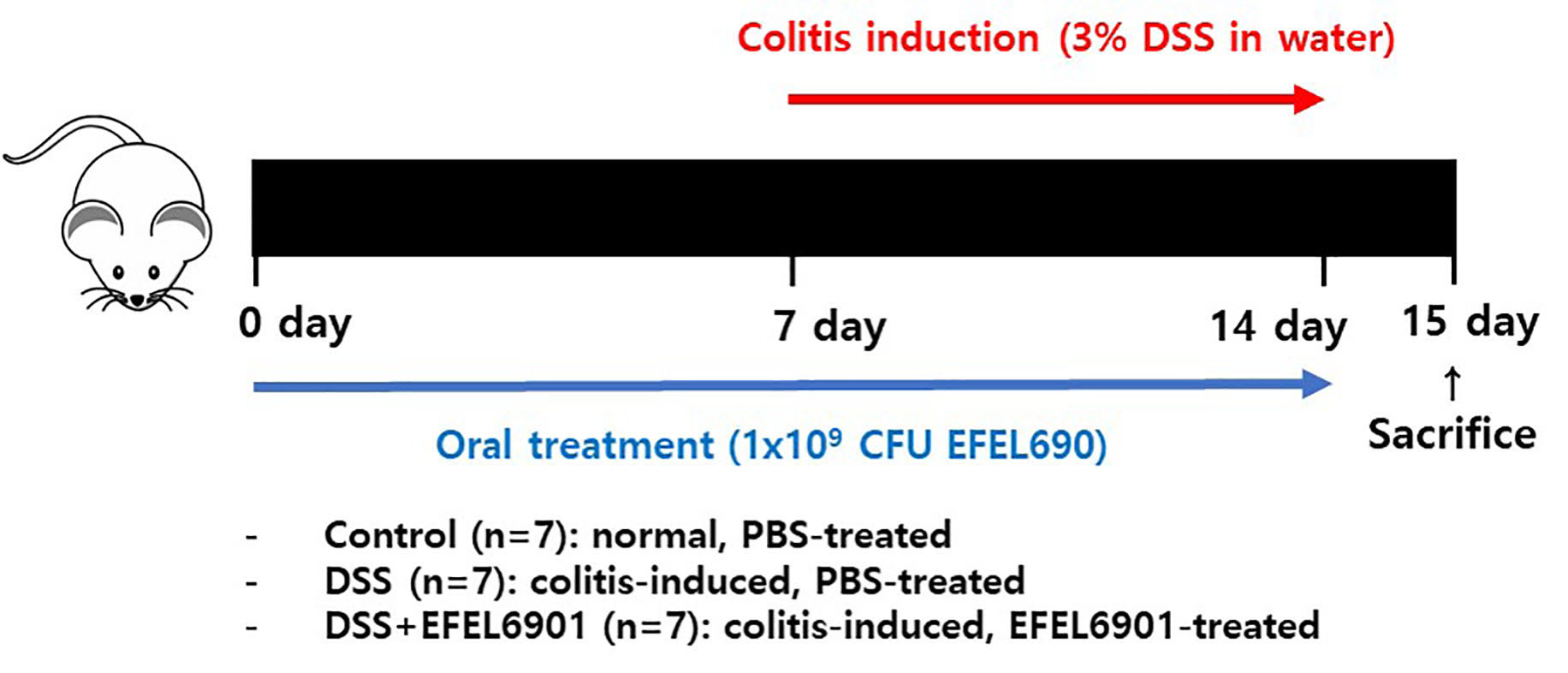

Supplement: Supplementary file 1 [file Image_1.JPEG]

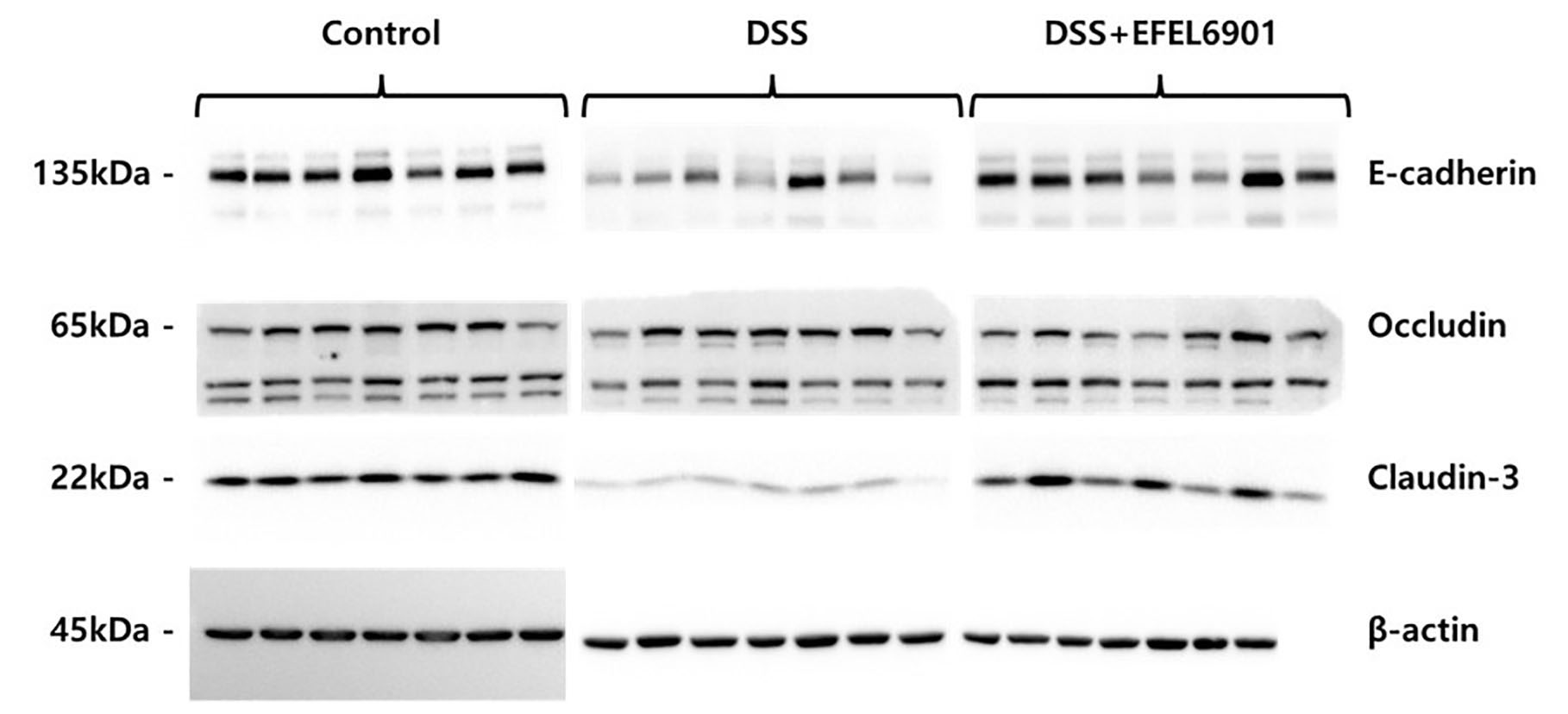

Supplement: Supplementary file 2 [file Image_2.JPEG]

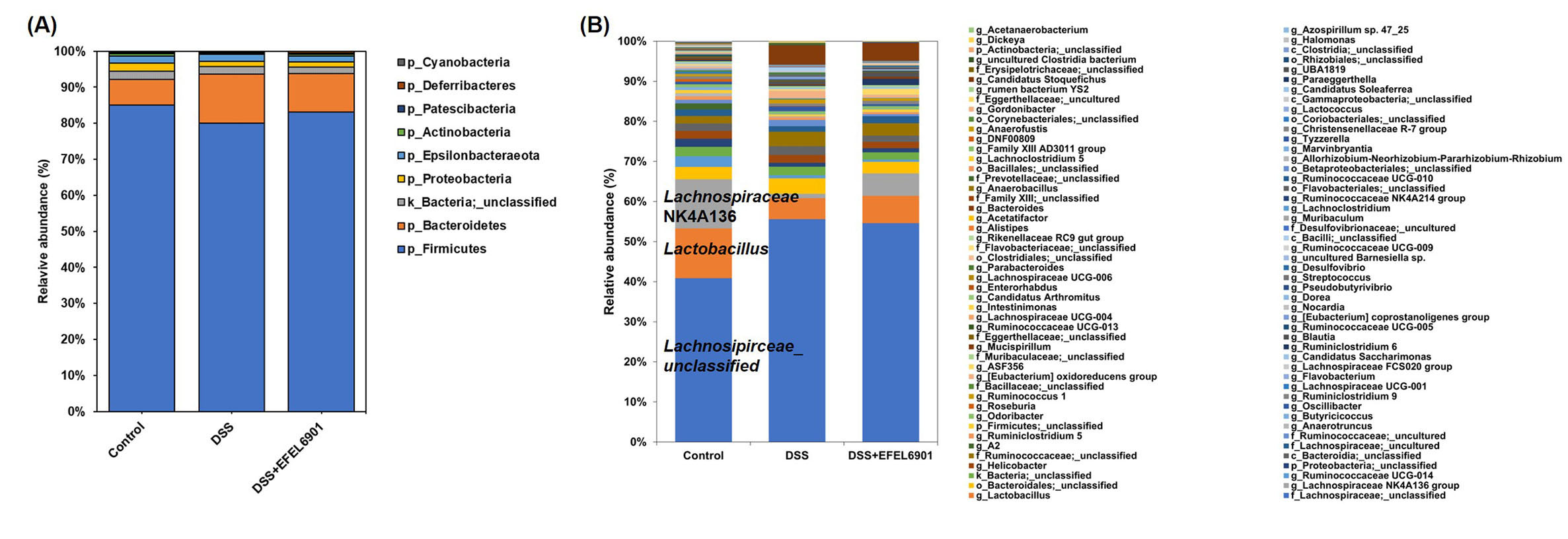

Supplement: Supplementary file 3 [file Image_3.JPEG]
