# Supplementary material for: Development of Anti-inflammatory Probiotic Limosilactobacillus reuteri EFEL6901 as Kimchi Starter: in vitro and In vivo Evidence
Source: Front Microbiol. 2021 Nov 25;12:760476. doi: 10.3389/fmicb.2021.760476 (PMC8656428; doi:10.3389/fmicb.2021.760476)
Supplement: Supplementary file 4 [file Table_1.DOCX]

Supplementary Material

Supplementary Table S1. Mouse-specific PCR primer sequences

| **Gene** | **Forward primer (5ʹ-3ʹ)** | **Reverse primer (5ʹ-3ʹ)** |
| --- | --- | --- |
| GAPDH | TTGTCTCCTGCGACTTCAACA | GCTGTAGCCGTATTCATTGTCATA |
| iNOS | ACCATGGAGCATCCCAAGTA | CCATGTACCAACCATTGAAGG |
| COX-2 | AGCATTCATTCCTCTACATAAGC | GTAACAACACTCACATATTCATACAT |
| TNF-α | ATGATCCGCGACGTGGAA | ACCGCCTGGAGTTCTGGA |
| IL-1β | GTTGACGGACCCCAAAAGAT | CACACACCAGCAGGTTATCA |
| IL-10 | GGACAACATACTGCTAACCGACTC | AAAATCACTCTTCACCTGCTCCAC |

Supplementary Table S2. List of strains used in this study

| **Species** | **Collections** | **Abbreviation** | **Culture condition** |
| --- | --- | --- | --- |
| *Limosilactobacillus reuteri* EFEL6901 | KACC 81105BP | EFEL6901 | 37 ℃, MRS |
| *Lactiplantibacillus plantarum* WCFS1 | ATCC BAA-793 | WCFS1 | 37 ℃, MRS |
| *Lacticaseibacillus rhamnosus* GG | KCTC 5033 | LGG | 37 ℃, MRS |
| *Limosilactobacillus reuteri* | ATCC 23272 | ATCC 23272 | 37℃, MRS |
| *Enterococcus faecalis* | KCCM 11729 | KCCM11729 | 37℃, BHI |
| *Listeria monocytogenes* | KCTC 3569 | KCTC3569 | 37℃, TSB |
| *Leuconostoc mesenteroides* DRC1506 | KCCM 11712P | DRC1506 | 30 ℃, MRS |

Supplementary Table S3. Calculated disease activity index (DAI) score (Jang et al., 2019)

| **Score** | **Weight loss (%)** | **Stool consistency** | **Blood in feces** |
| --- | --- | --- | --- |
| 0 | None | Normal | Negative  (No bleeding) |
| 1 | 1.0-5.0 | ­ | ­ |
| 2 | 5.0-10.0 | Loose stools | Positive  (Slight bleeding) |
| 3 | 10.0-15.0 | ­ | ­ |
| 4 | Over 15.0 | Watery diarrhea | Gross bleeding |

Supplementary Table S4. Criteria of histological scoring for evaluation of the inflammation in the large intestine (Wirtz et al., 2017).

| **Histological score** | **Inflammatory cell infiltration** | **Loss of crypt glands** |
| --- | --- | --- |
| 0 | Infrequent, ranged in normal | None |
| 1 | Mild increase of inflammatory cells in the lamina propria | Loss of glands, one third of mucosa |
| 2 | Moderate increase of inflammatory cells in the lamina propria | Loss of glands, two third of mucosa |
| 3 | Severe increase of inflammatory cells in the lamina propria and submucosa | Entirely loss of glands |
